# The use of continuous data versus binary data in MTC models: A case study in rheumatoid arthritis

**DOI:** 10.1186/1471-2288-12-167

**Published:** 2012-11-06

**Authors:** Susanne Schmitz, Roisin Adams, Cathal Walsh

**Affiliations:** 1 Department of Statistics, Trinity College Dublin, Dublin, Ireland; 2 National Centre for Pharmacoeconomics, , Dublin, Ireland

**Keywords:** Bayesian mixed treatment comparison models, Rheumatoid arthritis, Anti-TNF agents

## Abstract

**Background:**

Estimates of relative efficacy between alternative treatments are crucial for decision making in health care. When sufficient head to head evidence is not available Bayesian mixed treatment comparison models provide a powerful methodology to obtain such estimates. While models can be fit to a broad range of efficacy measures, this paper illustrates the advantages of using continuous outcome measures compared to binary outcome measures.

**Methods:**

Using a case study in rheumatoid arthritis a Bayesian mixed treatment comparison model is fit to estimate the relative efficacy of five anti-TNF agents currently licensed in Europe. The model is fit for the continuous HAQ improvement outcome measure and a binary version thereof as well as for the binary ACR response measure and the underlying continuous effect. Results are compared regarding their power to detect differences between treatments.

**Results:**

Sixteen randomized controlled trials were included for the analysis. For both analyses, based on the HAQ improvement as well as based on the ACR response, differences between treatments detected by the binary outcome measures are subsets of the differences detected by the underlying continuous effects.

**Conclusions:**

The information lost when transforming continuous data into a binary response measure translates into a loss of power to detect differences between treatments in mixed treatment comparison models. Binary outcome measures are therefore less sensitive to change than continuous measures. Furthermore the choice of cut-off point to construct the binary measure also impacts the relative efficacy estimates.

## Background

Meta-analysis has developed to be a widely used tool to combine trials evaluating the same intervention. This allows more powerful conclusions when a single study group is too small, and it fits naturally in the Bayesian framework where the inclusion of all available evidence into an analysis is anticipated [1]. A natural extension of a meta-analysis is a mixed treatment comparison (MTC) where more than two treatments are compared in a network of evidence. While direct evidence is preferable it is often not available due to ethical or financial reasons and MTC models provide a powerful tool to estimate relative efficacy among treatments which are not directly compared in a trial. A Bayesian approach to indirect comparison allows the inclusion of a wide range of evidence and is flexible to deal with increasingly complex evidence structures [2].

The variance of an indirect comparison is typically larger than the variance of a comparable direct comparison; the variance increases with every indirect link in the chain. It is therefore crucial to make the greatest use of available data.

Using a case study in rheumatoid arthritis this paper presents a MTC model on a range of efficacy measures illustrating the increased power of continuous outcome measures in such models compared to using binary measures. For the presentation of the analysis we follow the checklist suggested by Spiegelhalter et al. [3].

### Dichotomized outcome measures

Dichotomized outcome measures are widespread in medical research. The perceived advantage of simplicity in the interpretation comes at a cost, however [4]. The loss of information results in a loss of power to detect relationships, furthermore the type I error rate may be inflated [5] and there is a risk of underestimating the variance parameter. These issues have been discussed in the literature relating to a number statistical analyses [6-9].

In this work we demonstrate the consequences of dichotomized outcome measures in the context of MTC modelling. It has been pointed out that dichotomization can be used when designing trials to quantify a treatment effect [10,11]. This is the primary aim of many clinical trials aiming for license approval. The anticipated efficacy level is taken as a cut-off point and results allow a straightforward interpretation. Pharmacoeconomic assessments of healthcare interventions are now a formal component of decision making in many countries [12]. Evidence syntheses typically rely on published clinical trials to estimate the relative efficacy among alternative agents to inform decision making. This has created an additional use of clinical trials demonstrating treatment efficacy, for which dichotomized measures suffer from disadvantages compared to the underlying continuous effect measure. The loss of power due to dichotomization adds to the increased variance in indirect comparisons. This is especially problematic for MTCs (vs. pairwise meta-analysis), since they typically have large standard errors due to the indirect nature of the comparison.

### The intervention

Rheumatoid arthritis (RA) is a chronic, progressive and disabling auto-immune disease, causing swelling and damaging cartilage and bone around the joints. Any joint may be affected but it is commonly the hands, feet and wrists. Common symptoms are joint swelling, pain, morning joint stiffness, poor sleep, fatigue and weight loss [13].

Over the past decade, enhanced understanding of the molecular pathogenesis has led to the development of biologic agents that target specific parts of the immune system. These innovative treatments have altered the path and face of RA and outcomes for patients and society. Tumour necrosis factor alpha antagonists (anti-TNF-*α*) are the first of the biologic treatment groups used in RA. There are currently five anti-TNF agents licensed for RA in Europe; adalimumab, certolizumab, etanercept, golimumab and infliximab. All of these agents have demonstrated considerable efficacy in placebo controlled randomized controlled trials (RCTs) in patients who have had an inadequate response to conventional Disease Modifying Anti-Rheumatic Drugs (DMARDs) such as methotrexate (MTX) or sulphasalazine.

While there is a wealth of RCT evidence available for these agents compared to either placebo or conventional DMARDs, there are currently very limited head-to-head RCTs of anti-TNF agents. Despite this, some estimate of relative efficacy in order to inform choice of agent is needed. In the absence of head-to-head trials of relevant comparators, it is necessary to combine evidence from placebo controlled trials of different treatments and thereby derive an estimate of effect of one treatment against another. This can be broadly termed as mixed treatment comparison (MTC), an extension of meta-analysis. Different methodologies have been described for MTC; one such method uses Bayesian hierarchical models. Such models provide more flexibility than classical methods to include more data and handle more complex modelling structures [14].

### Aim of the analysis

The aim of this analysis is to demonstrate the advantage of using continuous measures in MTC models compared to using binary measures. In the present analysis a MTC model is fitted to estimate all pair-wise comparisons among the five TNF-*α*inhibitors and placebo for a range of outcome measures.

Nixon et al. [15] have developed a MTC model to fit the binomial American College of Rheumatology (ACR) outcome measure; it is possible to include trials with multiple treatment arms and to adjust for study level covariates. Jansen et al. [16] have presented a MTC model on the outcome of the Health Assessment Questionnaire (HAQ), which is measured on a continuous scale. However, they did not allow for multiple treatment arms or the inclusion of baseline characteristics. The present analysis extends Jansen’s methods to allow for these.

While binary measures are useful for demonstrating a certain level of efficacy in clinical trials, we will show that the loss of information when changing from the underlying continuous scale to the binary outcome measure results in a loss of power to detect differences between treatments in MTC analyses.

Fitting models for a continuous improvement measure and a discretised version thereof as well as for binary outcome measures and a continuous version of these illustrates the enhanced power to detect differences of continuous measures compared to binary measures.

The chosen outcome measures are based on the ACR criteria and the HAQ score. Details are described in the next section.

### Efficacy Measures

In order to estimate the relative efficacy between treatments one has to decide on a measure of disease activity and improvement. Commonly used measures in RA are the ACR criteria, the Disease Activity Score (DAS) and the HAQ score. Table 1 summarises the different measures of improvement. While other measures exist, they are outside the scope of this paper and are discussed elsewhere [17]. 

**Table 1:** Measuring improvement in RA: ACR (American College of Rheumatology); HAQ (Health Assessment Questionnaire); DAS28 (Disease Activity Score)

	***ACR***	***HAQ***	***DAS28***
continuous	meanACR	HAQ %-improvement	DAS28
	ACRhybrid		
binary	ACR 20	HAQ 20^∗^	
	ACR 50	HAQ 50^∗^	
	ACR 70		

^∗^Note: HAQ 20 and HAQ 50 are not validated response measures, they have been created here for the purpose of illustrating the loss of power in binary vs. continuous measures.

The ACR response criteria is a binary combination measure including the number of tender and swollen joints, patient’s global assessment, physician’s global assessment, pain, degree of disability and level of acute-phase reactant. In order to achieve an ACR 20, ACR 50 or ACR 70 result, an improvement of 20%, 50% or 70% respectively is required in the swollen and tender joint counts as well as in 3 of the 5 remaining dimensions [18,19].

Continuous measures based on the ACR criteria have been introduced including the meanACR and the ACR hybrid measure [20]. The meanACR measures the mean % improvement in the seven ACR core set measures. The ACR hybrid measure combines the ACR 20, ACR 50 and ACR 70 with the ACRmean. A patient’s ACR hybrid outcome is the same as the ACRmean, but restricted by his binary ACR response. For example, the outcome for a patient who is an ACR 20 responder, but not an ACR 50 responder is restricted to the interval [0.2, 0.5).

The HAQ score represents the result of a self-report questionnaire in which patients rate their ability to perform daily life activities such as washing one’s hair or getting in and out of a car. Values range from 0 to 3 in steps of 0.125, where high values indicate a more severe disease status. The improvement in HAQ score is measured on a continuous scale.

For the purpose of demonstrating the enhanced power of continuous measures in MTC models we have defined a discretised version of the HAQ score: HAQ 20 and HAQ 50. They are defined analogously to ACR 20 and ACR 50. A patient achieves a HAQ 20 outcome, if his HAQ score has improved by at least 20%; a 50% improvement is required for HAQ 50.

The DAS28 score is a combination measure on a continuous scale from 0 to 10 incorporating swollen joint count (*swollen28*), tender joint count (*tender28*) (out of 28 defined joints), an evaluation of the patients general health (*GH*) and the erythrocyte sedimentation rate (*ESR*). The score is obtained using the formula [21]:

(1) $$\begin{array}{ll} \text{DAS28}= & 0.56*\sqrt{\text{tender28}}+0.28*\sqrt{\text{swollen28}}\\ & +0.70*\ln(\text{ESR})+0.014*\text{GH} \end{array}$$

## Methods

### Data

A systematic literature review following the PRISMA method [22] was performed to identify trials meeting our inclusion criteria. The search included published studies up to and including October 2010 in PubMed, Embase and the Cochrane Database. Rheumatological inflammatory diseases other than RA, such as ankylosing spondylitis, psoriatic arthritis and connective tissue diseases were excluded from the search. The inclusion criteria were randomised controlled trials (RCTs), patients with established RA who have had an inadequate response to methotrexate (MTX) and who have been treated for at least 24 weeks (where 24 week data were not available, data within 6 weeks either before or after 24 weeks were used). Both monotherapy and combination therapy were included with an explicit term in the statistical model allowing for the additional effect of MTX. More details on the selection process can be found elsewhere [23].

The outcome measures chosen were those described in the previous section based on the ACR criteria, the HAQ score and the DAS28. Unfortunately the DAS28 was reported in too few trials to fit a MTC model (DAS28 was only reported in 6 trials, not representing each of the treatments). The total number of responders achieving ACR 20, ACR 50 and ACR 70 response and the mean improvement and standard deviations (SDs) for the continuous HAQ measure were extracted. Authors were contacted in cases where the required data were not reported. Where no access to the missing data was provided, the following methodology was applied: in cases where the mean was not reported, the median was used; in the absence of SDs, interquartile ranges (IQRs) were used to estimate SDs using a normal approximation, and, in the remaining cases, the maximum of clinical trial SDs was used. The doses of biological agents included are those included in the RCTs. Demographic data including age, gender, mean disease duration, baseline HAQ score and number of previous DMARDs were recorded.

### Statistical Model

**Figure 1:**
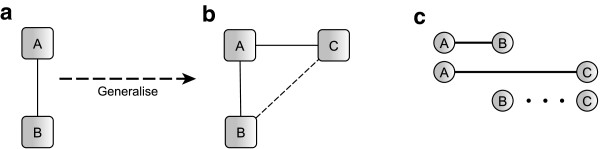
Generalisation from meta-analysis to mixed treatment comparison.

A Bayesian MTC model is fitted to the data for each of the outcome measures of interest (HAQ, HAQ 20, HAQ 50, ACR 20, ACR 50, ACR 70 and ACRcont). Such models simultaneously perform indirect comparisons between treatments that are not directly compared and allow estimation of all pair-wise comparisons. A network diagram represents the underlying evidence structure of such models. Figure 1 shows the generalisation of meta-analysis to the simplest case of an indirect comparison. Network diagrams consist of nodes representing the interventions included in the analysis and edges between nodes identify interventions which are directly compared in one or more trials. Dotted lines are used to indicate the indirect comparison of interest. For simplicity, these are often omitted when all comparisons for which no direct evidence is available is of interest.

In a simple meta-analysis evidence from a range of trials comparing the same two interventions A and B is combined yielding an overall A vs. B estimate (see evidence network in Figure 1(a)). The underlying methodology is well explored and used extensively in practice. The simplest case of a MTC occurs when this situation is extended to include a third intervention, drug C, which has also been compared to drug A in clinical trials (see Figure 1(b)). MTC modelling assumes that the combined evidence from A vs. B and A vs. C trials contains some information about the relative efficacy of B vs. C; in particular B vs. C is assumed to be the difference between A vs. B and A vs. C (see Figure 1(c)). MTC models can be fitted for all underlying evidence structures as long as the network diagram is connected. Combining evidence this way evokes the assumption of treatment exchangeability, a similarity assumption among the trials. The effect of B in the A vs. B trials is assumed to occur if C was replaced by B in the A vs. C trials. Unfortunately this assumption can only be tested in networks of closed loop design, where consistency measures can be obtained; methods are described elsewhere [24]. When this is not the case, great care has to be taken in selecting the trials for the analysis.

All models can be fitted in a Bayesian or in a classical framework. The Bayesian approach to MTC modelling is the most flexible, allowing the inclusion of a wide range of data and borrowing strength across the network ensures the optimal use of the data. Details on classical methods can be found elsewhere [25,26]. This analysis focuses on the Bayesian approach only.

In the remainder of this section the mathematical models for binary and continuous outcome measures are described beginning with simple meta-analytic models which are then extended to MTC models including baseline characteristics and other assumptions necessary for the RA model.

#### Simple Meta Analysis

In a simple meta analysis only two interventions are compared; evidence of a number of trials is combined to get an overall estimate for the difference in effect. The continuous mathematical model is the following [16]:

(2) $$\left\{\begin{array}{l} \Delta_{i}∼N(\delta_{i},\sigma_{i}^{2})\\ \delta_{i}∼N(d,\sigma_{\delta }^{2})\\ d∼[-,-]\;\sigma_{\delta }∼[-,-] \end{array}\right)$$

*Δ*_*i*_ is the observed relative treatment effect in study *i*; *σ*_*i*_the associated sampling error; *i*=1,…*N*, where *N* is the number of trials included in the analysis. The model assumes random effects, meaning the study specific true effects *δ*_*i*_are drawn from a normal distribution with mean *d* and between trial variance parameter $\sigma_{\delta }^{2}$. Prior distributions need to be specified for the basic parameter *d* and the between trial standard deviation *σ*_*δ*_. The choice of prior distribution is discussed in section “Prior distributions”, in this section we simply indicate which parameters require a prior distribution.

The binary counterpart to calculate odds ratios can be formulated as follows:

(3) $$\left\{\begin{array}{l} \mathrm{r.}c_{i}∼\text{Bin}(\mathrm{n.}c_{i},\mathrm{p.}c_{i});\;\mathrm{r.}t_{i}∼\text{Bin}(\mathrm{n.}t_{i},\mathrm{p.}t_{i})\\ \text{logit}(\mathrm{p.}c_{i})=\mu_{i}\\ \text{logit}(\mathrm{p.}t_{i})=\mu_{i}+\delta_{i}\\ \delta_{i}∼N(d,\sigma_{\delta }^{2})\\ d∼[-,-]\;\mu_{i}∼[-,-]\;\sigma_{\delta }∼[-,-] \end{array}\right)$$

A binomial likelihood is assumed for the number of patients and number of responders in control arm and treatment arm of each study; *n.c*_*i*_, *n.t*_*i*_, *r.c*_*i*_, *r.t*_*i*_respectively. The model calculates log odds ratios (LORs) for each study, *δ*_*i*_, for which random effects are assumed yielding an overall log odds ratio estimate *d* and a between trial standard deviation *σ*_*δ*_. Prior distributions need to be assigned to *d*, the logits in the control group *μ*_*i*_ and *σ*_*δ*_.

**Figure 2:**
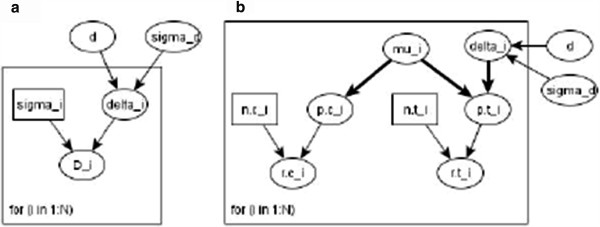
DAG for (a) continuous and (b) binary meta analysis model.

Figure 2 shows the directed acyclic graph (DAG) for (a) the continuous and (b) the binary meta-analysis model.

#### Extension to MTC

The models described in (2) and (3) for a simple meta analysis can be extended to a MTC model, which allows the estimation of relative efficacy among more than two interventions [14,16]. Mathematically this means the continuous model in equation (2) changes to:

(4) $$\left\{\begin{array}{l} \Delta_{i}∼N(\delta_{i,k,P},\sigma_{i}^{2})\\ \delta_{i,k,P}∼N(d_{k,P},\sigma_{\delta }^{2})\\ IC_{k,l}=d_{k,P}-d_{l,P}\;k\neq l\\ d_{k,P}∼[-,-]\;\sigma_{\delta }∼[-,-] \end{array}\right)$$

As before *Δ*_*i*_refers to the observed relative treatment effects of the comparison in study i with respective measure of variability *σ*_*i*_. *k* and *l* indicate the anti-TNF agent evaluated in the trials; 1 indicates adalimumab, 2 infliximab, 3 etanercept, 4 golimumab and 5 certolizumab. The baseline treatment is placebo, indicated by *P*. Assuming random effects, the study specific true effects are drawn from a normal distribution with a mean specific to the comparison; i.e. the effects of all trials comparing the same two interventions are drawn from the same normal distribution. The between trial variance parameter is assumed to be the same for all comparisons. Each comparison of drug k versus drug l, *IC*_*k*,*l*_, can be written in terms of basic parameters *d*_*k*,*P*_. Prior distributions are required for basic parameters and between trial standard deviation *σ*_*δ*_.

For the binary outcome measures the model in equation (3) extends to:

(5) $$\left\{\begin{array}{l} \mathrm{r.}c_{i}∼\text{Bin}(\mathrm{n.}c_{i},\mathrm{p.}c_{i});\;\mathrm{r.}t_{i}∼\text{Bin}(\mathrm{n.}t_{i},\mathrm{p.}t_{i})\\ \text{logit}(\mathrm{p.}c_{i})=\mu_{i}\\ \text{logit}(\mathrm{p.}t_{i})=\mu_{i}+\delta_{i,k,P}\\ \delta_{i,k,P}∼N(d_{k,P},\sigma_{\delta }^{2})\\ IC_{k,l}=d_{k,P}-d_{l,P}\;k\neq l\\ d_{k,P}∼[-,-]\;\mu_{i}∼[-,-]\;\sigma_{\delta }∼[-,-] \end{array}\right)$$

Again *n.c*_*i*_, *n.t*_*i*_, *r.c*_*i*_ and *r.t*_*i*_ refer to the number of patients and number of responders in the control and treatment arm of each study. The model calculates LORs *δ*_*i*,*k*,*P*_for each study, which are combined assuming random effects with a common mean for each baseline comparison *d*_*k*,*P*_. The between trial SD is assumed to be the same for all drugs. All other comparisons *IC*_*k*,*l*_can be estimated from the baseline parameters. Prior distributions need to be defined for *d*_*k*,*P*_, *μ*_*i*_ and *σ*_*δ*_.

#### Further extensions

For the use of the model for RA data three more adjustments have to be made. We want to allow for more than one treatment arm per study; adjustments have to be made for the concurrent treatment with MTX. Furthermore, we would like to model the improvement relative to baseline disease activity, since disease activity at baseline influences the effectiveness of the intervention [27]. Also, the results of this analysis provide the basis for an economic analysis, which requires the percentage improvement in HAQ score as input parameters.

A step by step approach is taken to include these assumptions into the model described in (4) for the continuous case and (5) for the binomial case.

As a first step the allowance for multiple treatment arms is included. Let index j refer to treatment arm j (*j*=1,…,*M*, where *M* is the total number of treatment arms in the analysis) and *s*(*j*) refers to the study of treatment arm *j*. The continuous mathematical model then takes the following form:

(6) $$\left\{\begin{array}{l} \Delta_{j}=\mathrm{\Delta .}t_{j}-\mathrm{\Delta .}c_{s(j)}\\ \Delta_{j}∼N(\delta_{j,k,P},\sigma_{j}^{2})\\ \delta_{j,k,P}=\delta_{s(j),k,P}\\ \delta_{s(j),k,P}∼N(d_{k,P},\sigma_{\delta }^{2})\\ IC_{k,l}=d_{k,P}-d_{l,P}\;k\neq l\\ d_{k,P}∼[-,-]\;\sigma_{\delta }∼[-,-] \end{array}\right)$$

For each treatment arm the observed effect relative to the comparator arm is calculated as *Δ*_*j*_, which as before has a normal distribution with measure of variability *σ*_*j*_. The model makes the assumptions that effects in treatment arms of the same study are constant, we therefore assume fixed effects for within study effects. This assumption is described in line 3 of equation (6). Between trials we assume random effects, as before and each comparison *IC*_*k*,*l*_ can be written in terms of basic parameters *d*_*k*,*P*_. Prior distribution need to be defined for basic parameters and between trial standard deviation *σ*_*δ*_.

This model takes the following form for binary outcome measures:

(7) $$\left\{\begin{array}{l} \text{r.}c_{s(j)}∼\text{Bin}(\text{n.}c_{s(j)},\mathrm{p.}c_{s(j)});\;\mathrm{r.}t_{j}∼\text{Bin}(\mathrm{n.}t_{j},\mathrm{p.}t_{j})\\ \text{logit}(\mathrm{p.}c_{s(j)})=\mu_{s(j)}\\ \text{logit}(\mathrm{p.}t_{j})=\mu_{s(j)}+\delta_{j,k,P}\\ \delta_{j,k,P}=\delta_{s(j),k,P}\\ \delta_{s(j),k,P}∼N(d_{k,P},\sigma_{\delta }^{2})\\ IC_{k,l}=d_{k,P}-d_{l,P}\;k\neq l\\ d_{k,P}∼[-,-]\;\mu_{s(j)}∼[-,-]\;\sigma_{\delta }∼[-,-] \end{array}\right)$$

Changes from equation (5) to (7) are analogous to changes in the continuous case. A LOR *δ*_*j*,*k*,*P*_ is obtained for each treatment arm; treatment arms of the same study are assumed to estimate the same treatment effect, hence line 4 in equation (7). Everything else remains the same.

In a next step we will extend the model to allow for the concurrent treatment with MTX. The effect due to MTX is assumed to be additive, meaning the relative effect between two arms where no MTX is given is the same as the relative effect between two arms where MTX is given in both arms:

(8) (A + MTX) vs. (B + MTX)=A vs. B

The continuous model takes the following form:

(9) $$\left\{\begin{array}{l} \Delta_{j}=\mathrm{\Delta .}t_{j}-\mathrm{\Delta .}c_{s(j)}\\ \Delta_{j}∼N(\delta_{j,k,P},\sigma_{j}^{2})\\ \delta_{j,k,P}=\alpha_{s(j),k,P}+\beta_{s(j)}(\mathrm{I.}t_{j}-\mathrm{I.}c_{s(j)})\\ \alpha_{s(j),k,P}∼N(a_{k,P},\sigma_{\alpha }^{2})\\ \beta_{s(j)}=b\\ IC_{k,l}=a_{k,P}-a_{l,P}\;k\neq l\\ a_{k,P}∼[-,-]\;b∼[-,-]\\ \sigma_{\alpha }∼[-,-] \end{array}\right)$$

The difference to the previous model described in equation (6) is the splitting of treatment effect *δ*_*j*,*k*,*P*_into a part representing the effect due to the drug of interest (in the RA case the anti-TNF effect) *α*_*s*(*j*),*k*,*P*_and a part explaining the effect which is due to the concurrent treatment with MTX, *β*_*s*(*j*)_. *I.t*_*i*_ and *I.c*_*s*(*i*)_ are indicator variables indicating whether MTX was given in treatment and comparator group. By multiplying the *β*_*s*(*i*)_ with the difference of the indicator the assumptions of an additive effect as explained above is implemented. In this way, only treatment arms, where MTX is given in either the treatment arm or in the respective control arm inform parameter *β*_*s*(*j*)_. As previously the effect in arms of the same study is assumed constant, hence *β*_*j*_=*β*_*s*(*j*)_ and *α*_*j*,*k*,*P*_=*α*_*s*(*j*),*k*,*P*_ (this line has been omitted in the equation for simplicity; the effect is incorporated in line 3 of equation 9). Between trials random effects are assumed for *α*_*s*(*j*),*k*,*P*_ and fixed effects for *β*_*s*(*j*)_. One could just as well assume random effects for *β*_*s*(*j*)_; but the RA data does not provide sufficient evidence to inform a between trial variability for b. All comparisons *IC*_*k*,*l*_ can be expressed in terms of basic parameters *a*_*k*,*P*_. Prior distributions need to be specified for the basic parameters, for *b* and for the between trial standard deviation *σ*_*α*_. The binary version of this model is mathematically described as follows:

(10) $$\left\{\begin{array}{l} \mathrm{r.}c_{s(j)}∼\text{Bin}(\mathrm{n.}c_{s(j)},\mathrm{p.}c_{s(j)});\;\mathrm{r.}t_{i}∼\text{Bin}(\mathrm{n.}t_{j},\mathrm{p.}t_{j})\\ \text{logit}(\mathrm{p.}c_{s(j)})=\mu_{s(j)}+\beta_{s(j)}*\mathrm{I.}c_{s(j)}\\ \text{logit}(\mathrm{p.}t_{j})=\mu_{s(j)}+\beta_{s(j)}*\mathrm{I.}t_{s(j)}+\alpha_{s(j),k,P}\\ \alpha_{s(j),k,P}∼N(a_{k,P},\sigma_{\alpha }^{2})\\ \beta_{s(j)}=b\\ IC_{k,l}=a_{k,P}-a_{l,P}\;k\neq l\\ a_{k,P}∼[-,-]\;\mu_{s(j)}∼[-,-]\;b∼[-,-]\\ \sigma_{\alpha }∼[-,-] \end{array}\right)$$

Again, changes follow analogously to changes made in the continuous case. In a last step we want to extend the model such that the drug effect *α*_*s*(*j*),*k*,*P*_depends on baseline disease activity. To model relative percentage improvement *α* is modelled as a multiplier to the HAQ score at baseline *λ*_*i*_. All other aspects of model (9) remain unchanged.

(11) $$\left\{\begin{array}{l} \Delta_{j}=\mathrm{\Delta .}t_{j}-\mathrm{\Delta .}c_{s(j)}\\ \Delta_{j}∼N(\delta_{j,k,P},\sigma_{j}^{2})\\ \delta_{j,k,P}=\alpha_{s(j),k,P}*\lambda_{j}+\beta_{s(j)}(\mathrm{I.}t_{j}-\mathrm{I.}c_{s(j)})\\ \alpha_{s(j),k,P}∼N(a_{k,P},\sigma_{\alpha }^{2})\\ \beta_{s(i)}=b\\ IC_{k,l}=a_{k,P}-a_{l,P}\;k\neq l\\ a_{k,P}∼[-,-]\;b∼[-,-]\;\sigma_{\alpha }∼[-,-] \end{array}\right)$$

The binomial measures selected for this study are by definition relative to baseline disease activity, since the cut-off between case and no case is *a* % improvement relative to baseline. It is therefore not necessary to adjust the model defined in equation 10 further. For completeness, we will introduce the concept of meta-regression, which allows for the adjustment for baseline characteristics in the analysis. The resulting model is:

(12) $$\left\{\begin{array}{l} \mathrm{r.}c_{s(j)}∼\text{Bin}(\mathrm{n.}c_{s(j)},\mathrm{p.}c_{s(j)});\;\mathrm{r.}t_{j}∼\text{Bin}(\mathrm{n.}t_{j},\mathrm{p.}t_{j})\\ \text{logit}(\mathrm{p.}c_{s(j)})=\mu_{s(j)}+\beta_{s(j)}*\mathrm{I.}c_{s(j)}\\ \text{logit}(\mathrm{p.}t_{j})=\mu_{s(j)}+\beta_{s(j)}*\mathrm{I.}t_{s(j)}+\alpha_{s(j),k,P}+\gamma *\lambda_{s(j)}\\ \alpha_{s(j),k,P}∼N(a_{k,P},\sigma_{\alpha }^{2})\\ \beta_{s(j)}=b\\ IC_{k,l}=a_{k,P}-a_{l,P}\;k\neq l\\ a_{k,P}∼[-,-]\;\mu_{s(j)}∼[-,-]\;b∼[-,-]\\ \sigma_{\alpha }∼[-,-]\;\gamma ∼[-,-] \end{array}\right)$$

*γ* refers to the regression parameter for the baseline HAQ score.

Models described in equations 10 and 11 enable the estimation of relative efficacy of a number of treatments while allowing for multiple treatment arms, concurrent treatment with additional medication and dependence of treatment effect on baseline disease activity for both continuous and binary outcome measures. In the continuous case differences in efficacy are estimated; odds ratios are estimated in the binary case. Using methods by Warn et al. [28] the model can be modified to calculate other measures, such as risk ratios.

**Figure 3:**
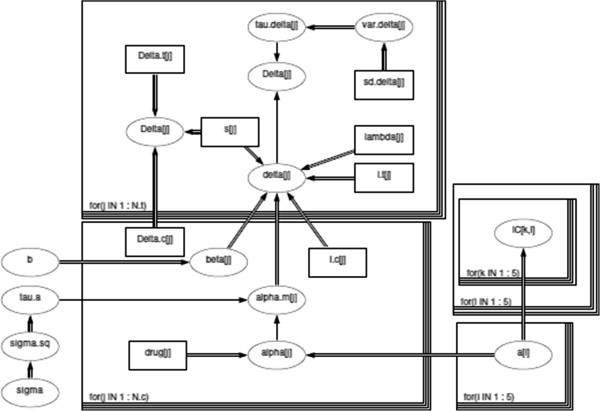
**DAG for continuous MTC model in equation (**11**).** Square shaped nodes represent constants, oval shaped nodes are either random or logical. A random dependency between two nodes can be found where a single arrow connects both nodes, a double arrow shows that there is a logical relationship. Loops are represented by boxes.

This model has been developed by Nixon et al. [15] for the binary case. Figure 3 shows the DAG for the final MTC models described by equations (11). 

### Prior distributions

The models described above require prior distributions for the baseline treatment effect, for the effect due to MTX, for the between trial standard deviation and in the binomial models for the log odds of response in the control group and the meta-regression parameter. For the analysis we have chosen vague priors for all of these as described below. The prior distribution in a Bayesian analysis represents the knowledge about the parameter prior to the analysis; in the absence of prior knowledge vague prior distributions intend to cover a wide area of plausible values, such that little information is entered into the analysis. For the treatment effect parameters *d* in model (2) and (3), *d*_*k*,*P*_ in models (4)-(7), for *a*_*k*,*P*_and *b* in (9) - (12), for the log odds in the control group of the binomial models *μ*_*i*_ as well as for *γ*we have chosen a normal distribution centred at no treatment effect 0 with a very large variance:

(13) $$\begin{array}{c} d\;∼N(0,10000)\\ d_{k,P}∼N(0,10000)\\ a\;∼N(0,10000) \end{array}$$

$$\begin{array}{c} b\;∼N(0,10000)\\ \mu_{i}\;\;\;∼N(0,10000)\\ \gamma \;∼N(0,10000) \end{array}$$

The same prior can be used for both continuous and binary models, since the risk difference and the log odds ratios take values on the real line where 0 represents no difference. The priors do not favour any of the drugs and allows for a wide range of likely values in favour for each. This is a widely used vague prior on treatment effects.

A uniform distribution was chosen for the between trial standard deviation parameter *σ*_*δ*/*α*_, as proposed by Gelman [29].

(14) $$\sigma_{\delta /\alpha }∼\text{dunif}(0,2)$$

To test whether the range of the uniform prior was chosen appropriately the analysis has been rerun using wider ranges (*dunif*(0,5) and *dunif*(0,7)) and estimates did not change.

### Computation/ Software

The MTC models were fitted in WinBUGs, a MCMC software using Gibbs sampling [30]. The DAG in Figure 3 is drawn in WinBUGs. Square shaped nodes represent constants; oval shaped nodes are either random or logical. A random dependency between two nodes can be found where a single arrow connects both nodes, a double arrow shows that there is a logical relationship. Loops are represented by boxes. The complete code and input data is provided in Additional file 1. Computational feasability allowed for a large number of iterations. Each model discarded 50,000 burnin iterations and was run with 100,000 iterations and two chains. Convergence was analysed using CODA; the effective sample size was checked, and visual inspection of the autocorrelation and the chains confirmed convergence.

## Results and discussion

### Data

**Figure 4:**
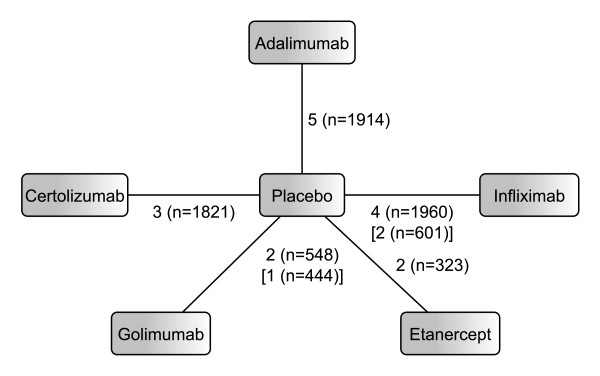
**Network diagram for RA analysis.** Edges are labelled with the number of studies and the total number of patients included in these studies. Numbers in square brackets refer to HAQ evidences where this differs from ACR evidence.

The systematic literature review identified sixteen RCTs meeting our inclusion criteria. Figure 4 shows the network of available evidence. Table 2 summarises the data extracted from the trials, baseline demographics can be found in Additional file 2.

**Table 2:** **Trial Data: Number of patients N; improvement in HAQ score *Δ *HAQ; number of ACR20, ACR50, ACR70 responders; HAQ score at baseline HAQ**_***base***_**; + indicates additional treatment with MTX**

***Trial***	***Arm***	***N***	***ΔHAQ*(*SD*)**	***ACR20***	***ACR50***	***ACR70***	***HAQ***_***base***_
Weinblatt et al. [31]	P+	62	0.27 (0.6)	9	5	3	1.64
	Ada+	69	0.54 (0.6)	33	22	7	1.52
	Ada+	67	0.62 (0.6)	45	37	18	1.55
	Ada+	73	0.59 (0.5)	48	31	14	1.55
Keystone et al. [32]	P+	200	0.24 (0.5)	59	19	5	1.45
	Ada+	207	0.56 (0.5)	131	81	43	1.44
	Ada+	212	0.60 (0.5)	129	87	37	1.48
Van de Putte et al. [33]	P	110	0.07 (0.5)	21	9	2	1.88
	Ada	112	0.39 (0.6)	44	23	11	1.88
	Ada	106	0.29 (0.6)	38	20	9	1.88
	Ada	103	0.49 (0.5)	55	36	19	1.84
	Ada	113	0.38 (0.6)	52	25	14	1.83
Miyasaka [34]	P	87	-0.1 (0.6)	12	5	1	1.39
	Ada	87	0.2 (0.5)	25	14	9	1.57
	Ada	91	0.2 (0.6)	40	22	11	1.64
	Ada	87	0.4 (0.6)	44	28	13	1.77
Kim at el. [35]	P+	63	0.2 (0.5)	23	9	5	1.3
	Ada+	65	0.5 (0.6)	40	28	14	1.4
Maini et al. [36]	P+	88	0.3 (0.5)†	18	7	0	1.8
	Inf+	86	0.3 (0.5)*†*	45	22	7	1.8
	Inf+	86	0.5 (0.5)*†*	47	25	9	1.8
	Inf+	87	0.5 (0.6)*†*	51	26	15	1.8
	Inf+	81	0.4 (0.5)*†*	49	21	9	1.5
Westhoven et al.[37]	P+	363	-	87	33	16	1.5
	Inf+	360	-	199	110	48	1.5
	Inf+	361	-	205	119	54	1.5
Zhang et al. [38]	P+	86	0.45 (-)	42	22	12	1.6
	Inf+	87	0.76 (-)	66	38	20	1.6
Schiff et al. [39]	P+	110	-	49	22	10	1.8
	Inf+	165	-	98	61	40	1.7
Moreland et al. [40]	P	80	0.03 (-)	9	4	1	1.7
	Eta	76	0.58 (-)	39	18	7	1.7
	Eta	78	0.62 (-)	46	31	12	1.6
Weinblatt et al. [41]	P+	30	0.4 (-)	8	1	0	1.5
	Eta+	59	0.7 (-)	42	23	9	1.5
Keystone et al. [42]	P+	133	0.13red* (0.4)*‡*	37	18	7	1.25*
	Gol	133	0.13* (0.7)*‡*	47	26	15	1.38*
	Gol+	89	0.38* (0.5)*‡*	53	33	18	1.38*
	Gol+	89	0.5* (0.5)*‡*	53	29	16	1.38*
Kay et al. [43]	P+	35	-	13	2	0	1.3
	Gol+	35	-	21	13	3	1.7
	Gol+	34	-	19	10	6	1.8
Keystone et al. [44]	P+	199	0.18 (-)	27	15	6	1.7
	Cert+	393	0.60 (-)	231	146	84	1.7
	Cert+	390	0.63 (-)	237	156	79	1.7
Smolen et al. [45]	P+	127	0.14 (0.5)	11	4	1	1.6
	Cert+	246	0.5 (0.5)	141	80	39	1.6
	Cert+	246	0.5 (0.5)	142	81	26	1.6
Fleischmann et al. [46]	P	109	-0.07 (0.4)*†*	10	4	0	1.6
	Cert	111	0.39 (0.7)*†*	51	25	9	1.4

Ada=adalimumab; Inf=infliximab; Eta=etanercept; Gol=golimumab, Cert=certolizumab, P=placebo. *Median; *‡*estimated from IQR; *†*data provided by authors following request.

The MTC models described previously are fit to the data; the continuous model for the improvement in HAQ score and the binary model for the ACR 20, ACR 50 and ACR 70 outcome measures.

The aim of this paper is to demonstrate the increased power of continuous outcome measures to detect differences in MTC models compared to binary measures. To explore this hypothesis the HAQ score was discretised to a binary outcome measure and the ACR criteria were transformed into a continuous measure; the resulting measures were analysed in addition to the trial reported outcome measures and results compared. The rationale for doing this was that comparing HAQ and ACR outcomes is not comparing like with like; they measure quite different aspects of disease and additional significant findings using the HAQ score could be because of this rather than there being any effect of a continuous vs. a binary outcome measure. Estimating a discrete version of the HAQ and a continuous measure of the ACR allows us to compare binary and continuous outcomes while the measure is kept fixed.

### Discretised HAQ: HAQ 20 and HAQ 50

For the purpose of comparing continuous and binary outcome measures based on the same data, the HAQ 20 and HAQ 50 measures defined previously are estimated based on the continuous HAQ improvement. These discretised HAQ outcomes are not validated outcome measures for RA and are therefore not reported in the trials.

The trials report mean and standard deviation of HAQ improvement. The number of HAQ 20 and HAQ 50 responders in each trial arm was estimated as follows. For each trial arm, the HAQ improvement of 1000 patients was generated by reference to normal distribution with mean and SD given from the data in that group. From these patients the proportion of HAQ 20 and HAQ 50 responders was calculated and applied to the number of patients in each trial arm. Data is summarised in Table 3.

**Table 3:** Input Data for discretised HAQ score and continuous ACR: Number of patients N; number of HAQ20 and HAQ50 responders; continuous ACR response ACRcont; + indicates additional treatment with MTX

***Trial***	***Arm***	***N***	***HAQ20***	***HAQ50***	***ACRcont (SD)***
Weinblatt et al. [31]	P+	62	30	12	0.08 (0.22)
	Ada+	69	45	24	0.35 (0.20)
	Ada+	67	47	26	0.46 (0.22)
	Ada+	73	52	26	0.42 (0.21)
Keystone et al. [32]	P+	200	95	32	0.13 (0.22)
	Ada+	212	141	81	0.42 (0.21)
	Ada+	207	154	82	0.41 (0.21)
Van de Putte et al. [33]	P	110	29	4	0.09 (0.20)
	Ada	112	47	16	0.31 (0.19)
	Ada	106	60	19	0.30 (0.19)
	Ada	103	57	20	0.38 (0.22)
	Ada	113	60	21	0.33 (0.20)
Miyasaka [34]	P	87	23	8	0.07 (0.17)
	Ada	87	37	10	0.28 (0.19)
	Ada	91	39	16	0.33 (0.20)
	Ada	87	48	18	0.37 (0.21)
Kim et al. [35]	P+	63	28	10	0.18 (0.27)
	Ada+	65	43	24	0.42 (0.22)
Maini et al. [36]	P+	88	40	9	0.09 (0.19)
	Inf+	86	40	12	0.35 (0.19)
	Inf+	86	54	19	0.36 (0.20)
	Inf+	87	52	23	0.38 (0.21)
	Inf+	81	48	20	0.37 (0.19)
Westhoven et al. [37]	P+	363	-	-	0.12 (0.23)
	Inf+	360	-	-	0.37 (0.20)
	Inf+	361	-	-	0.38 (0.21)
Zhang et al. [38]	P+	86	52	28	0.27 (0.31)
	Inf+	87	61	39	0.46 (0.20)
Schiff et al. [39]	P+	110	-	-	0.22 (0.29)
	Inf+	165	-	-	0.41 (0.22)
Moreland et al. [40]	P	80	25	8	0.06 (0.16)
	Eta	76	47	26	0.34 (0.19)
	Eta	78	53	31	0.40 (0.21)
Weinblatt et al. [41]	P+	30	16	8	0.10 (0.17)
	Eta+	59	45	29	0.43 (0.19)
Keystone et al. [42]	P+	133	49	13	0.14 (0.25)
	Gol	133	56	29	0.30 (0.20)
	Gol+	89	51	24	0.40 (0.22)
	Gol+	89	60	31	0.39 (0.21)
Kay et al. [43]	P+	35	-	-	0.14 (0.20)
	Gol+	35	-	-	0.39 (0.20)
	Gol+	34	-	-	0.38 (0.21)
Keystone et al. [44]	P+	199	83	35	0.07 (0.20)
	Cert+	393	263	146	0.40 (0.22)
	Cert+	390	249	137	0.41 (0.22)
Smolen et al. [45]	P+	127	46	10	0.04 (0.14)
	Cert+	246	163	66	0.38 (0.21)
	Cert+	246	155	67	0.38 (0.20)
Fleischmann et al. [46]	P	109	19	2	0.04 (0.14)
	Cert	111	60	34	0.33 (0.19)

Ada=adalimumab; Inf=infliximab; Eta=etanercept; Gol=golimumab, Cert=certolizumab, P=placebo.

### Continuous ACR: ACRcont

While the continuous ACR measures were proposed by the Americal College of Rheumatology [20], they were not assessed in any of the trials. Therefore a continuous ACR measure ACRcont is generated based on the ACR 20, ACR 50 and ACR 70 outcomes reported in the trials. This enables us to compare the outcomes of the binary measures with those resulting from the continuous scale. ACRcont takes a value between 0 and 1 representing the percentage improvement in the dimensions which are combined for the binary ACR criteria.

The trial data allows the categorisation of patients into non-responders (group 1) (patients not achieving an ACR 20 response), patients achieving ACR 20 but not ACR 50 response (group 2), patients achieving ACR 50 but not ACR 70 response (group 3) and ACR 70 responders (group 4). Assuming a mean ACRcont response *m*_*i*_ for each group of patients these numbers are used to generate the mean and SD of ACRcont for each trial arm:

(15) $$\mathrm{Mean}(\text{ACRcont})=\frac{1}{N}\left(\overset{4}{\underset{i=1}{\sum }}N_{i}\cdot m_{i}\right)$$

where *N*_*i*_ refers to the number of patients in each group and $N=\overset{4}{\underset{i=1}{\sum }}N_{i}$.

(16) $$\begin{array}{c} \text{sd}(\text{ACRcont})=\frac{1}{N}\sqrt{\overset{4}{\underset{i=1}{\sum }}N_{i}\cdot {\left(m_{i}-\text{Mean}(\text{ACRcont})\right)}^{2}} \end{array}$$

Patient level data from a RA cohort allowed the estimation of *m*_*i*_ for the four groups [47]. Mean ACRcont response can be based on either continuous ACR measure described previously, depending upon which underlying measure we are trying to recreate. For the main analysis we are using the meanACR measure; results based on the ACRhybrid are discussed in the sensitivity analysis. Table 4 summarizes the mean ACRcont responses for the four groups based on both outcome measures. Improvement in the core sets is measured by a drop in score; therefore overall improvement is restricted by an upper bound of 100%. To achieve a symmetric measuring scale overall worsening has also been restricted to -100% in each of the criteria, as proposed elsewhere [20]. 

**Table 4:** Mean improvement estimated from patient level data

	***group 1***	***group 2***	***group 3***	***group 4***
meanACR	0.17	0.41	0.57	0.72
ACRhybrid	0.10	0.41	0.59	0.77
Control Group	0.00	0.35	0.60	0.85

The patient level estimates are based on patients receiving biologic treatment; estimates can therefore be applied to the treatment arms of the studies. Unfortunately no patient level data representing the control groups is available to us. Given the lack of further information the analysis assumes interval midpoints as mean ACRcont estimates for the control groups. Group 1 is assumed to have a response in [-1, 0.2), group 2 in [0.2, 0.5), group 3 in [0.5, 0.7) and the response of group 4 lies in [0.7, 1]. The interval for non responders is very broad covering a wide range of extreme values. The baseline analysis therefore assumes a conservative mean value of 0.00 for this group. This yields the control group estimates in Table 4. Alternative scenarios assuming the same mean response for the control group as estimated for the treatment group are analysed in the sensitivity analysis. The estimated input data for the continuous ACR model based on meanACR is summarised in Table 3.

### Efficacy Results

The models calculate the relative efficacy among all anti-TNF agents and placebo. Relative efficacy for the continuous measures (HAQ improvement and ACRcont) was measured as difference in improvement. Relative improvement was modelled as a multiplier to baseline HAQ score. Odds ratios were calculated for the binomial measures ACR 20, ACR 50, ACR 70 and HAQ 20 and HAQ50. Thirteen trials were included for the HAQ analysis and sixteen for the ACR analysis.

**Figure 5:**
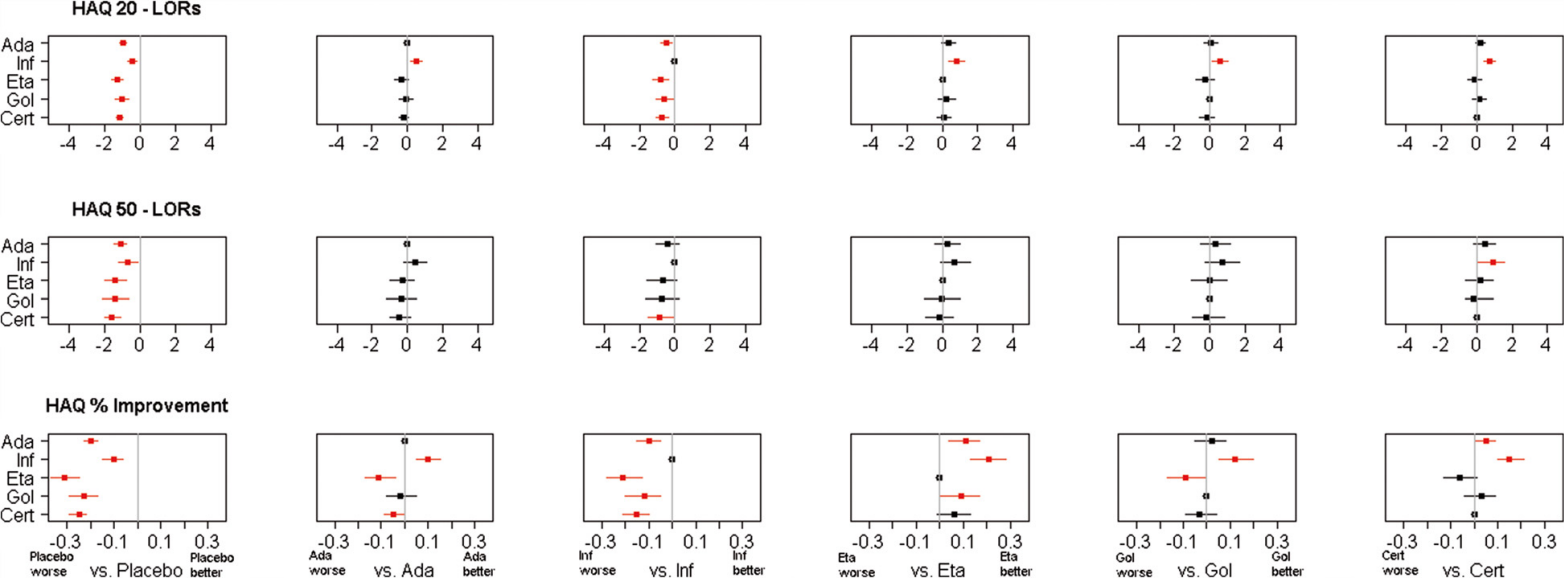
**Forest Plot of HAQ results.** Plots pairwise LORs for HAQ 20 and HAQ 50 outcome and improvement differences for % improvement in HAQ score of anti-TNF agents against placebo and one another. Red colour indicates significant differences at the 80% level.

**Figure 6:**
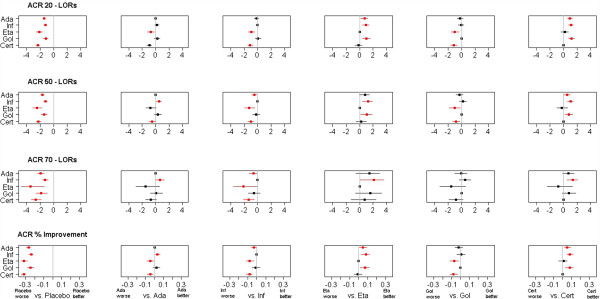
**Forest Plot of ACR results.** Plots pairwise LORs for ACR 20, ACR 50 and ACR 70 outcome and improvement differences for ACRcont of anti-TNF agents against placebo and one another. Red colour indicates significant differences at the 80% level.

The MTC results are summarised in forest plots; in Figure 5 for the HAQ measures and in Figure 6 for the ACR measures. Binomial results are plotted on the log scale. Plots show mean and 80% credible intervals to summarize the posterior distribution for the relative efficay between anti-TNF agents; significant differences on the 80% level are marked in red. The 80% level was chosen to shift away from the misunderstood acceptance-rejection dichotomy of random signifiance levels [23]. For additional information, Tables 5 and 6 provide the exact means and interval endpoints; significant differences on the 80% level are marked with a “∗”. 

**Table 5:** Results from HAQ analysis: Mean estimate with 80% credible intervals for each pair-wise comparison

***Comparison***	***HAQ***	***HAQ20***	***HAQ50***
Ada vs P	0.20 (0.17,0.23)*	2.6 (2.2,3.1)*	3.1 (2.1,4.4)*
Inf vs P	0.10 (0.06,0.15)*	1.6 (1.2,2.1)*	2.0 (1.2,3.5)*
Eta vs P	0.31 (0.25,0.37)*	3.6 (2.6,5.2)*	4.1 (2.2,7.7)*
Gol vs P	0.23 (0.17,0.29)*	2.8 (2.0,4.1)*	4.2 (1.9,8.8)*
Cert vs P	0.25 (0.22,0.29)*	3.3 (2.7,4.0)*	5.0 (3.0,7.6)*
Inf vs Ada	-0.10 (-0.15,-0.05)*	0.6 (0.4,0.8)*	0.7 (0.3,1.3)
Eta vs Ada	0.11 (0.04,0.17)*	1.4 (0.9,2.1)	1.3 (0.7,2.7)
Eta vs Inf	0.21 (0.13,0.28)*	2.3 (1.4,3.6)*	2.0 (0.9,4.9)
Gol vs Ada	0.02 (-0.05,0.08)	1.1 (0.7,1.6)	1.4 (0.6,3.3)
Gol vs Inf	0.12 (0.05,0.20)*	1.8 (1.1,2.8)*	2.1 (0.8,5.4)
Gol vs Eta	-0.09 (-0.17,0.00)*	0.8 (0.5,1.3)	1.0 (0.4,2.7)
Cert vs Ada	0.05 (0.00,0.09)*	1.2 (0.97,1.6)	1.6 (0.8,2.8)
Cert vs Inf	0.15 (0.10,0.21)*	2.0 (1.5,2.9)*	2.4 (1.1,4.7)*
Cert vs Eta	-0.06 (-0.13,0.01)	0.9 (0.6,1.3)	1.2 (0.5,2.5)
Cert vs Gol	0.03 (-0.04,0.09)	1.1 (0.8,1.7)	1.2 (0.4,2.7)
*σ*	0.03 (0.01,0.05)	0.1 (0.0,0.3)	0.4 (0.1,0.6)

Significant results are marked with ∗. Outcome measures are % improvement for continuous HAQ and ORs for HAQ 20 and HAQ 50. Ada=adalimumab; Inf=infliximab; Eta=etanercept; Gol=golimumab, Cert=certolizumab, P=placebo, *σ*=between trial SD.

**Table 6:** Results from ACR analysis: Mean estimate with 80% credible intervals for each pair-wise comparison

***Comparison***	***ACR 20***	***ACR 50***	***ACR 70***	***ACRcont***
Ada vs P	4.2 (3.4,5.3)*	5.6 (4.3,7.2)*	7.0 (4.3,10.9)*	0.27 (0.25,0.29)*
Inf vs P	3.5 (2.8,4.4)*	3.4 (2.7,4.4)*	3.7 (2.3,5.5)*	0.24 (0.22,0.27)*
Eta vs P	8.8 (5.5,13.9)*	12.1 (5.7,23.2)*	30.9 (4.2,123.8)*	0.32 (0.28,0.35)*
Gol vs P	3.3 (2.3,4.8)*	4.2 (2.7,6.5)*	6.5 (2.8,13.3)*	0.25 (0.21,0.28)*
Cert vs P	10.6 (8.0,14.1)*	9.5 (6.7,13.5)*	14.3 (6.8,27.0)*	0.32 (0.30,0.35)*
Inf vs Ada	0.8 (0.6,1.1)	0.6 (0.4,0.9)*	0.5 (0.3,1.0)*	-0.03 (-0.06,0.00)*
Eta vs Ada	2.1 (1.2,3.4)*	2.2 (0.96,4.2)	4.4 (0.6,19.0)	0.05 (0.01,0.09)*
Eta vs Inf	2.5 (1.5,4.1)*	3.5 (1.5,6.9)*	8.3 (1.1,37.7)*	0.08 (0.03,0.12)*
Gol vs Ada	0.8 (0.5,1.2)	0.8 (0.4,1.2)	0.9 (0.4,2.2)	-0.02 (-0.06,0.02)
Gol vs Inf	0.9 (0.6,1.5)	1.2 (0.7,2.0)	1.7 (0.7,4.1)	0.01 (-0.04,0.05)
Gol vs Eta	0.4 (0.2,0.7)*	0.3 (0.2,0.8)*	0.2 (0.0,1.8)	-0.07 (-0.12,-0.02)*
Cert vs Ada	2.5 (1.8,3.6)*	1.7 (1.1,2.6)*	2.1 (0.9,4.7)	0.05 (0.02,0.09)*
Cert vs Inf	3.0 (2.1,4.3)*	2.8 (1.8,4.3)*	3.8 (1.7,8.3)*	0.08 (0.05,0.12)*
Cert vs Eta	1.2 (0.7,2.0)	0.8 (0.4,1.8)	0.5 (0.1,3.6)	0.01 (-0.04,0.05)
Cert vs Gol	3.2 (2.0,5.1)*	2.3 (1.3,3.9)*	2.2 (0.8,6.4)	0.08 (0.03,0.12)*
*σ*	0.2 (0.0,0.4)	0.2 (0.0,0.4)	0.5 (0.0,0.8)	0.03 (0.01,0.04)

Significant results are marked with ∗. Outcome measures are ORs for ACR 20, ACR 50 and ACR 70 and % improvement for ACRcont. Ada=adalimumab; Inf=infliximab; Eta=etanercept; Gol=golimumab, Cert=certolizumab, P=placebo, *σ*=between trial SD.

The key point is that all differences between agents detected by the binary HAQ measures are also detected by the continuous HAQ improvement measure, while not all differences detected in the continuous HAQ model are detected using the binary HAQ data; the differences detected by the binary HAQ measures are subsets of the differences detected by the continuous HAQ improvement measure. This is also demonstrated in the ACR analysis. It illustrates the point made that a continuous measure of effect has a greater power to detect a difference between treatments in an evidence synthesis.

## Conclusion

This analysis illustrates the enhanced sensitivity to change of continuous measures in MTC models compared to using binary outcome measures, which was recently highlighted for epidemiological studies [48]. While binary measures work well for demonstrating a certain level of response, which is the primary aim of many clinical trials, the information lost when categorizing the underlying continuous response can have significant impact on the results of a mixed treatment comparison.

MTC models are often utilized when faced with a choice of agents rather than for demonstrating efficacy. In the above application, for example, it is already known that all of the anti-TNF agents provide improvement in the treatment of RA. What remains is to determine whether some of these agents work better than others. Therefore it would be of interest to know whether an agent provides a 30% improvement or a 60% improvement. When looking at the ACR 20 outcome, no difference would be seen between the two response rates.

The above analyses have shown two things. Firstly, the enhanced power to detect differences of a continuous measure as opposed to a binary measure was shown for both, the HAQ and the ACR measures. Secondly, the choice of cut-off level (e.g. 20%, 50% or 70%) has shown to have a strong impact on the results. Different significant results were detected when choosing different cut-offs; even the sequence of treatments when ordering from best to worse changes.

It may be argued that binary outcomes are clear and easy to interpret. However, where binary outcome measures are required, results based on continuous measures can be transformed subsequently using cut-off points, [49].

From a clinical point of view these results show that a MTC model based on continuous outcome measures provides greater precision of estimates of efficacy. This is of great benefit when carrying out an economic evaluation.

### Sensitivity Analysis

The trials included for the analysis were conducted over a time period of 10 years. The model was extended to a meta-regression to include potential confounding parameters such as duration of disease, number of previous DMARDs and year of publication. None of these were found to have a significant impact and therefore were not included in the main analysis.

Different prior distributions on the between trial variance parameter allowing for a wider range of values were tested, but did not alter the conclusions. Varying the precision parameter of the normal prior distributions for the remaining parameters between 1,000 and 100,000 did not alter the results either.

The continuous ACR response underlying the binary ACR 20, ACR 50 and ACR 70 measures is estimated using the mean ACRcont response for each of the four responder groups. For the baseline analysis meanACR was used as the underlying continuous effect. In a sensitivity analysis a model was fit using the ACR hybrid as the underlying effect. This did not alter the results much, however, adalimumab was not found to be superior to infliximab in this scenario. There was substantial uncertainty regarding the mean ACRcont response in the control arms, as no patient level data was available. An additional model has been fit assuming the mean response in each group to be the same for treatment arms and control arms. While this did influence the biologic treatment effect versus control, the relative efficacy between biologic treatments was not affected much. Again, these models did not detect a difference between adalimumab and infliximab. Outcomes of the various MTCs can be accessed in Additional file 3.

The scale chosen for dichotomized outcome measures has been shown to influence outcomes of MTC models [50]. In addition to the OR scale we therefore also fitted an analysis based on risk ratios (RRs). The OR model can be adapted to estimate RRs making a few changes, for further details see Schmitz et al. [23]. The results can be accessed in Additional file 4; the findings of increased power of continuous outcomes are confirmed.

### Limitation

Doses across treatments and treatment arms varied, which has not been accounted for in the analysis. Generalising the model to include a meta-regression for dose would raise difficulties of comparability; doses across treatments are hard to compare as well as within one treatment when the same dose is given, but with different frequency.

The analysis only includes RCT evidence. However, there exists a large body of observational data collected via registries and open label studies. The inclusion of such evidence is possible within this framework, helping to reduce uncertainty further [51].

Patient level data allowed the estimation of mean ACR response within each of the responder groups. While this provided some additional information towards estimating the continuous ACR response measure, it did not replace the full information lost. The estimated ACRcont measure is still subject to high uncertainty. Patient level data of the RCTs included in the analysis would allow the exact calculation of the continuous ACR measure.

The evidence network has a star design, which does not allow testing of the assumption of treatment exchangeability. Baseline characteristics across the trials were compared and and the model was extended to a meta-regression to explain the potential influence of differing demographics.

This paper confirms the effectiveness of anti-TNF agents in the treatment of RA. The study illustrates the enhanced ability to detect differences between treatments when using continuous measures and shows the high dependency of MTC outcomes on the cut-off level when using binary data.

Rather than steering away from binary measures, we would like to advocate the additional reporting of underlying continuous effect measures in clinical trials to facilitate further analyses. The importance of the involvement of statisticians in the choice of clinical measures has been recently highlighted [10].

## Competing interests

The authors declare that they have no competing interests.

## Authors’ contributions

SS is responsible for the study concept and design, designed and implemented the MTC models and participated in the systematic review and data extraction. RR is responsible for the study concept and design and carried out the systematic review and data extraction. CW oversaw the design and implementation of the MTC models. All authors contributed to the interpretation of findings. All authors read and approved the final manuscript.

## Pre-publication history

The pre-publication history for this paper can be accessed here:

http://www.biomedcentral.com/1471-2288/12/167/prepub

## Supplementary Material

Additional file 1 **Bugs code.** Additional file providing the WinBUGs code and input data for the MTC model. Click here for file

Additional file 2 **Baseline Demographics.** Mean Baseline Demographics of randomized controlled trials for anti-TNF agents. Click here for file

Additional file 3 **Sensitivity Analysis (1).** Additional file providing outcomes of the sensitivity analysis conducted on the continuous ACR measure. Click here for file

Additional file 4 **Sensitivity Analysis (2).** Additional file providing outcomes of the sensitivity analysis conducted on the risk ratio scale. Click here for file
