# Supplementary material for: The use of continuous data versus binary data in MTC models: A case study in rheumatoid arthritis
Source: BMC Med Res Methodol. 2012 Nov 6;12:167. doi: 10.1186/1471-2288-12-167 (PMC3576322; doi:10.1186/1471-2288-12-167)
Supplement: Additional file 1 — Bugs code. Additional file providing the WinBUGs code and input data for the MTC model. [file 1471-2288-12-167-S1.docx]

***Bugs code for continuous model:***

model{

for (j in 1: N.t){ ***### Calculate precision***

var.delta[j] <- sd.delta[j]*sd.delta[j]

tau.delta[j] <- 1/var.delta[j]}

for (j in 1: N.t){ ***### Model***

Delta[j] <- Delta.t[j] - Delta.c[s[j]]

Delta[j] ~ dnorm(delta[j],tau.delta[j])

delta[j]<-alpha[s[j]]*lambda[j] + beta[s[j]]*(I.t[j]-I.c[s[j]])

}

for (i in 1:N.c){ ***### Random effects for biologic treatment effect***

alpha[i] ~ dnorm(a[drug[i]], tau.a)

beta[i] <- b}

tau.a <- 1 / sigma.sq

sigma.sq <- sigma * sigma

***### Prior Distributions***

sigma ~ dunif(0,2)

b ~ dnorm(0,0.0001)

for( i in 1 : 5 ) {a[i] ~ dnorm(0,0.0001)}

***### Indirect estimates***

for( k in 1 : 5 ) {

for( l in 1 : 5 ) {

IC[k,l] <- a[k] - a[l] } }

}

***Bugs code for binary model:***

model{

***### Model***

for (i in 1:N.c){

r.c[i] ~ dbin(p.c[i],n.c[i])

logit(p.c[i])<-mu[i] + beta[i]*I.c[i]}

for (i in 1:N.t){

r.t[i] ~ dbin(p.t[i],n.t[i])

logit(p.t[i])<- mu[s[i]] + beta[s[i]]*I.t[i] + alpha[s[i]] + gamma*(lambda[s[i]]-lambda.bar)}

lambda.bar<-mean(lambda[])

for (i in 1:N.c){ ***### Random effects for biologic treatment effect***

alpha[i] ~ dnorm(a[drug[i]],tau.alpha)

beta[i] <- b }

tau.alpha<- 1/sigma.sq

sigma.sq <- sigma*sigma

***### Prior Distributions***

for (i in 1:N.c){

mu[i] ~ dnorm(0,1.0E-6)}

b ~ dnorm(0,1.0E-6)

for (i in 1:5){

a[i] ~ dnorm(0,1.0E-6)}

sigma ~ dunif(0,2)

gamma ~ dnorm(0,1.0E-6)

***### Indirect estimates***

for (k in 1:5){

for(l in 1:5){

IC[k,l] <- a[k] - a[l]}}

}

***Input Data***

%improvement in HAQ score

list(N.c=13, N.t=29, *### Number of control and treatment arms*

s=c(1,1,1,2,2,3,3,3,3,4,4,4,5,6,6,6,6,7,8,8,9,10,10,10,11,11,12,12,13*), ### respective control arm to treatment arm*

I.c=c(1,1,0,0,1,1,1,0,1,1,1,1,0), *### Indicator for MTX in control arm*

I.t=c(1,1,1,1,1,0,0,0,0,0,0,0,1,1,1,1,1,1,0,0,1,0,1,1,1,1,1,1,0), *### Indicator for MTX in treatment arm*

lambda=c(1.52,1.55,1.55,1.44,1.48,1.88,1.88,1.84,1.83,1.57,1.64,1.77,1.4,1.8,1.8,1.8,1.5,1.6,1.7,1.6,1.5,1.38,1.38,1.38,1.7,1.7,1.6,1.6,1.43), *### HAQ score at baseline*

Delta.c=c(0.27,0.24,0.07,-0.1,0.2,0.3,0.45,0.03,0.4,0.13,0.18,0.14,-0.07), *### HAQ improvement control*

Delta.t=c(0.54,0.62,0.59,0.56,0.6,0.39,0.29,0.49,0.38,0.2,0.2,0.4,0.5,0.3,0.5,0.5,0.4,0.76,0.58,0.62,0.7,0.13,0.38,0.5,0.6,0.63,0.5,0.5,0.39), *### HAQ improvement treatment*

drug=c(1,1,1,1,1,2,2,3,3,4,5,5,5), *### indicator for biologic drug*

sd.delta=c(0.1,0.11,0.1,0.05,0.05,0.07,0.08,0.07,0.07,0.08,0.09,0.09,0.09,0.08,0.08,0.08,0.08,0.1,0.11,0.11,0.15,0.07,0.06,0.06,0.06,0.06,0.05,0.05,0.08) *### standard error of improvement*)

ACRcont***

list(

s=c(1,1,1,2,2,3,3,3,3,4,4,4,5,6,6,6,6,7,7,8,9,10,10,11,12,12,12,13,13,14,14,15,15,16),

*### Respective control arm to treatment arm*

I.c=c(1,1,0,0,1,1,1,1,1,0,1,1,1,1,1,0), *### Indicator for MTX in control arm*

I.t=c(1,1,1,1,1,0,0,0,0,0,0,0,1,1,1,1,1,1,1,1,1,0,0,1,0,1,1,1,1,1,1,1,1,0), *### Indicator for MTX treatment*

N.t=34, N.c=16, *### Number of control and treatment arms*

drug=c(1,1,1,1,1,2,2,2,2,3,3,4,4,5,5,5), *### indicator for biologic drug*

Delta=c(0.27,0.38,0.34,0.28,0.27,0.22,0.21,0.29,0.24,0.21,0.27,0.30,0.24,0.26,0.27,0.29,0.28,0.25,

0.26,0.19,0.19,0.29,0.34,0.32,0.16,0.26,0.25,0.24,0.23,0.33,0.34,0.34,0.34,0.29), *### ACR improvement*

sd.delta=c(0.04,0.04,0.04,0.02,0.02,0.03,0.03,0.03,0.03,0.03,0.03,0.03,0.04,0.03,0.03,0.03,0.03,

0.02,0.02,0.04,0.03,0.03,0.03,0.04,0.03,0.03,0.03,0.05,0.05,0.02,0.02,0.02,0.02,0.02) *### standard error of improvement* )

inits <- list( list( b=-0.1, a=c(-0.1,-0.1,-0.1,-0.1,-0.1), sigma=0.9, alpha=c(-0.1,-0.1,-0.1,-0.1,-0.1,-0.1,-0.1,-0.1,-0.1,-0.1,-0.1,-0.1,-0.1)),

list( b=0.1, a=c(0.1,0.1,0.1,0.1,0.1), sigma=1.1, alpha=c(0.1,0.1,0.1,0.1,0.1,0.1,0.1,0.1,0.1,0.1,0.1,0.1,0.1)))

HAQ 20 and HAQ 50

List(N.c =13, N.t = 29, *### Number of control and treatment arms*

n.c = c(62,200,110,87,63,88,86,80,30,133,199,127,109), ### *Number of patients in control arms*

I.c = c(1,1,0,0,1,1,1,0,1,1,1,1,0), *### Indicator for MTX in control arm*

s = c(1,1,1,2,2,3,3,3,3,4,4,4,5,6,6,6,6,7,8,8,9,10,10,10,11,11,12,12,13),

I.t = c(1,1,1,1,1,0,0,0,0,0,0,0,1,1,1,1,1,1,0,0,1,0,1,1,1,1,1,1,0), *### Indicator for MTX in treatment arm*

n.t = c(69, 67, 73, 207, 212, 112, 106, 103, 113, 87, 91, 87, 65, 86, 86, 87, 81, 87, 76, 78, 59, 133, 89, 89, 393, 390, 246, 246, 111), *### number of patients in treatment arm*

drug = c(1,1,1,1,1,2,2,3,3,4,5,5,5), *### indicator for biologic drug*

r.c = c(30,95,29,23,28,40,52,25,16,49,83,46,19), *### number of HAQ20 responders in control*

r.t =c(45,47,52,141,154,47,60,57,60,37,39,48,43,40,54,52,48,61,47,53,45,56,51,60,263,249, 163,155,60) *### number of HAQ20 responders in treatment arm*)

*### HAQ 50 responders control and treatment*

r.c = c(12,32,4,8,10,9,28,8,8,13,35,10,2)

r.t = c(24,26,26,81,82,16,19,20,21,10,16,18,24,12,19,23,20,39,26,31,29,29,24,31,146, 137,66,67,34)

*### Initial values*

inits <- list (list (a=c(0.1,0.1,0.1,0.1,0.1), b=0.1, sigma=0.8, mu=c(0.1,0.1,0.1,0.1,0.1,0.1,0.1,0.1,0.1,0.1,0.1,0.1,0.1), alpha=c(0.1,0.1,0.1,0.1,0.1,0.1,0.1,0.1,0.1,0.1,0.1,0.1,0.1)),

list(a=c(-0.1,-0.1,-0.1,-0.1,-0.1), b=-0.1, sigma=1.2, mu=c(-0.1,-0.1,-0.1,-0.1,-0.1,-0.1,-0.1,-0.1,-0.1,-0.1,-0.1,-0.1,-0.1), alpha=c((-0.1,-0.1,-0.1,-0.1,-0.1,-0.1,-0.1,-0.1,-0.1,-0.1,-0.1,-0.1,-0.1)))

ACR 20, ACR 50 and ACR 70

list( N.c = 16, N.t = 34, *### Number of control and treatment arms*

n.c = c(62,200,110,87,63,88,363,86,110,80,30,133,35,199,127,109), ### *Number of patients in control arms*

I.c = c(1,1,0,0,1,1,1,1,1,0,1,1,1,1,1,0), *### Indicator for MTX in control arm*

s = c(1,1,1,2,2,3,3,3,3,4,4,4,5,6,6,6,6,7,7,8,9,10,10,11,12,12,12,13,13,14,14,15,15,16),

*### Respective control arm to treatment arm*

I.t = c(1,1,1,1,1,0,0,0,0,0,0,0,1,1,1,1,1,1,1,1,1,0,0,1,0,1,1,1,1,1,1,1,1,0), *### Indicator for MTX in treatment arm*

n.t = c(69,67,73,207,212,112,106,103,113,87,91,87,65,86,86,87,81,360,361,87,165,76,78,59,133, 89,89, 35,34,393,390,246,246,111), *### number of patients in treatment arm*

drug = c(1,1,1,1,1,2,2,2,2,3,3,4,4,5,5,5), *### indicator for biologic drug*

r.c = c(9,59,21,12,23,18,87,42,46,9,8,37,13,27,11,10), *### number of ACR20 responders in control*

r.t = c(33,45,48,131,129,44,38,55,52,25,40,44,40,45,47,51,49,199,205,66,98,39, 46,42, 47, 53, 53, 21,19, 231, 237,141,142,51) *### number of ACR20 responders in treatment*)

*### ACR 50 responders in control and treatment arms:*

r.acr50.c = c(5,19,9,5,9,7,33,22,22,4,1,18,2,15,4,4)

r.acr50.t = c(22,37,31,81,87,23,20,36,25,14,22,28,28,22,25,26,21,110,119,38,61,18,31,23,26,33, 29,13,10,146,156,80,81,25)

*### ACR 70 responders in control and treatment arms:*

r.c =c(3,5,2,1,5,0,16,12,10,1,0,7,0,6,1,0) r.t = c(7,18,14,43,37,11,9,19,14,9,11,13,14,7,9,15,9,48,54,20,40,7,12,9,15,18,16,3,6,84,79, 39,26,9)

** Adjustments to code for ACRcont: ACR already is a % improvement; therefore no baseline disease parameter is included. Also delta is already calculated from delta.c and delta.t, this line*
