# Supplementary material for: The use of continuous data versus binary data in MTC models: A case study in rheumatoid arthritis
Source: BMC Med Res Methodol. 2012 Nov 6;12:167. doi: 10.1186/1471-2288-12-167 (PMC3576322; doi:10.1186/1471-2288-12-167)
Supplement: Additional file 2 — Baseline Demographics. Mean Baseline Demographics of randomized controlled trials for anti-TNF agents. [file 1471-2288-12-167-S2.docx]

Mean Baseline Demographics of Randomised Controlled Trials for anti-TNF agents

| **Study** | **Total number in the trial arms used (N)** | **Age (Yrs)** | **Disease Duration (Yrs)** | **Number of Previous DMARDs** | **Baseline HAQ Score** | **Dose of MTX (mg)** |
| --- | --- | --- | --- | --- | --- | --- |
| Weinblatt et al. 2003 (ARMADA) | 271 | 56 | 12 | 3 | 1.6 | 17 |
| Keystone et al. 2004 | 619 | 56 | 11 | 2 | 1.5 | 17 |
| Van de Putte et al. 2004 | 544 | 53 | 11 | 4 | 1.9 | n/a |
| Miyasaka et al. 2008 (CHANGE) | 352 | 55 | 7 | n/a | 1.6 | n/a |
| Kim et al. 2007 | 128 | 49 | 7 | n/a | 1.4 | 16 |
| **Mean Demographic for adalimumab trials** |  | **55** | **10** | **3** | **1.6** | **17** |
| Maini et al. 1999 (ATTRACT) | 428 | 53 | 8 | 3 | 1.7 | 15 |
| Westhovens et al. 2006 (START)* | 1,084 | 52 | 7 | n/a | 1.5 | 15 |
| Zhang et al. 2006 | 173 | 48 | 8 | n/a | n/a | n/a |
| Schiff et al. 2008 (ATTEST)* | 275 | 49 | 8 | n/a | 1.7 | 16 |
| **Mean Demographics for infliximab trials** |  | **52** | **8** | **3** | **1.6** | **15** |
| Moreland et al. 1999 | 234 | 52 | 12 | 3 | 1.7 | n/a |
| Weinblatt et al. 1999 | 89 | 50 | 13 | 3 | 1.5 | 19 |
| **Mean Demographics for etanercept trials** |  | **52** | **12** | **3** | **1.6** | **19** |
| Keystone et al. 2009 | 444 | 51 | 6 | n/a | 1.3 | 15 |
| Kay et al. 2008* | 104 | 53 | 7 | n/a | 1.5 | n/a |
| **Mean Demographics for golimumab trials** |  | **52** | **6** | **n/a** | **1.4** | **15** |
| Keystone et al. 2008 (RAPID 1) | 982 | 52 | 6 | 1 | 1.7 | 14 |
| Smolen et al. 2009 (RAPID 2) | 619 | 52 | 6 | 1 | 1.6 | 13 |
| Fleischmann et al. 2009 (FAST4WARD) | 220 | 54 | 10 | 2 | 1.5 | n/a |
| **Mean Demographics for certolizumab pegol trials** |  | **52** | **7** | **1** | **1.6** | **13** |

*These studies were excluded for the analysis of the HAQ data due to lack of data.
