# Supplementary material for: The use of continuous data versus binary data in MTC models: A case study in rheumatoid arthritis
Source: BMC Med Res Methodol. 2012 Nov 6;12:167. doi: 10.1186/1471-2288-12-167 (PMC3576322; doi:10.1186/1471-2288-12-167)
Supplement: Additional file 3 — Sensitivity Analysis (1). Additional file providing outcomes of the sensitivity analysis conducted on the continuous ACR measure. [file 1471-2288-12-167-S3.docx]

Sensitivity analysis on ACRcont

The estimation of ACRcont based on ACR 20, ACR 50 and ACR 70 is subject to a substantial amount of uncertainty. The RCTs only report the number of ACR 20, ACR 50 and ACR 70 responders, the additional information from the underlying continuous cause is lost. Patient level data from a RA cohort allowed us to estimate the mean ACR response of each of these responder groups for patients treated with biologic agents. The baseline analysis assumed meanACR as underlying continuous response and given the lack of further knowledge, interval midpoints for the control arms. In addition we have analysed three further scenarios:

1. **ACRhybrid – MP:** ACRhybrid as underlying continuous response and midpoints for control groups.
2. **mACR – mACR:** mean ACR is taken as underlying response; assumes the mean ACRcont response in the control group to follow the same distribution as in the treatment group.
3. **ACRhyb – ACRhyb:** As in 2), but taking ACRhybrid to be the underlying continuous measure.

The following table summarises the results of the ACRcont mixed treatment comparison based on baseline assumptions and the additional three scenarios:

| Comparison (A vs. B) | Baseline | ACRhybrid - MP | mACR – mACR | ACRhyb - ACRhyb |
| --- | --- | --- | --- | --- |
|  | **Mean (80% CrI)** | **Mean (80% CrI)** | **Mean (80% CrI)** | **Mean (80% CrI)** |
| Ada vs. Placebo | **0.27 (0.25,0.29)*** | **0.25 (0.23,0.27)*** | **0.13 (0.11,0.15)*** | **0.13 (0.11,0.15)*** |
| Inf vs. Placebo | **0.24 (0.22,0.27)*** | **0.22 (0.20,0.25)*** | **0.12 (0.10,0.14)*** | **0.12 (0.10,0.14)*** |
| Eta vs. Placebo | **0.32 (0.28,0.35)*** | **0.30 (0.26,0.34)*** | **0.17 (0.14,0.21)*** | **0.17 (0.14,0.21)*** |
| Gol vs. Placebo | **0.25 (0.21,0.28)*** | **0.23 (0.19,0.27)*** | **0.12 (0.09,0.16)*** | **0.12 (0.09,0.16)*** |
| Cert vs. Placebo | **0.32 (0.30,0.35)*** | **0.30 (0.28,0.33)*** | **0.17 (0.15,0.19)*** | **0.17 (0.15,0.19)*** |
| Inf vs. Ada | **-0.03 (-0.06,0.00)*** | **-0.02 (-0.06,0.01)** | **-0.01 (-0.04,0.02)** | **-0.01 (-0.04,0.02)** |
| Eta vs. Ada | **0.05 (0.01,0.09)*** | **0.06 (0.01,0.10)*** | **0.04 (0.01,0.09)*** | **0.04 (0.00,0.08)*** |
| Eta vs. Inf | **0.08 (0.03,0.12)*** | **0.08 (0.03,0.12)*** | **0.05 (0.01,0.09)*** | **0.05 (0.01,0.09)*** |
| Gol vs. Ada | **-0.02 (-0.06,0.02)** | **-0.02 (-0.07,0.02)** | **-0.01 (-0.05,0.03)** | **-0.01 (-0.05,0.03)** |
| Gol vs. Inf | **0.01 (-0.04,0.05)** | **0.00 (-0.05,0.05)** | **0.00 (-0.04,0.04)** | **0.00 (-0.04,0.04)** |
| Gol vs. Eta | **-0.07 (-0.12,-0.02)*** | **-0.08 (-0.13,-0.02)*** | **-0.05 (-0.09,0.00)*** | **-0.05 (-0.09,0.00)*** |
| Cert vs. Ada | **0.05 (0.02,0.09)*** | **0.06 (0.02,0.09)*** | **0.04 (0.01,0.07)*** | **0.04 (0.01,0.07)*** |
| Cert vs. Inf | **0.08 (0.05,0.12)*** | **0.08 (0.04,0.12)*** | **0.05 (0.02,0.08)*** | **0.05 (0.02,0.08)*** |
| Cert vs. Eta | **0.01 (-0.04,0.05)** | **0.00 (-0.05,0.05)** | **0.00 (-0.04,0.04)** | **0.00 (-0.04,0.04)** |
| Cert vs. Gol | **0.08 (0.03,0.12)*** | **0.08 (0.03,0.13)*** | **0.05 (0.01,0.09)*** | **0.05 (0.01,0.09)*** |
| sigma | **0.03 (0.01,0.04)** | **0.03 (0.02,0.04)** | **0.03 (0.01,0.04)** | **0.03 (0.01,0.04)** |

Outcomes of scenario 1 are very similar to the baseline results. However, adalimumab does not appear superior to infliximab, since the credible intervals crosses 0. Both, scenario 2 and 3 give very similar results. Assuming the same mean response for the patient groups in control arm as in treatment arms results in overall lower efficacy estimates for the biologic treatments versus control (scenario 2 and 3). Outcomes of all continuous analyses represent %-improvement based on a fixed efficacy measure. The estimates of scenario 2 and 3 appear quite low when compared to the estimates of the continuous HAQ analysis; assuming interval midpoints for the control group hence appears to be a more realistic assumption. The indirect estimates of one biologic agent against another are similar to the baseline analysis and scenario 1. Again, the credible interval of infliximab versus adalimumab includes 0.

In the binary case adalimumab appeared superior to infliximab in the ACR 50 and ACR 70 analysis, not in the ACR 20 analysis. The fact that there are a lot more patients in group 1 and group 2 (non-responder and ACR 20 responder that are not ACR 50 responder) than in group 3 and group 4 (patients that are at least ACR 50 responders) could explain the fact that this difference was not detected in the three additional scenarios. The overall difference of adalimumab and infliximab appears bigger in the 20% cut-off than in the overall effect. This highlights the impact of cut-off level on the results.
