# Supplementary material for: The use of continuous data versus binary data in MTC models: A case study in rheumatoid arthritis
Source: BMC Med Res Methodol. 2012 Nov 6;12:167. doi: 10.1186/1471-2288-12-167 (PMC3576322; doi:10.1186/1471-2288-12-167)
Supplement: Additional file 4 — Sensitivity Analysis (2). Additional file providing outcomes of the sensitivity analysis conducted on the risk ratio scale. [file 1471-2288-12-167-S4.docx]

Sensitivity analysis Risk Ratios

The scale of outcome measures for binary data has shown to affect the outcome of a MTC analysis. An analysis based on risk ratios (RRs) in addition to the analysis based on ORs is therefore conducted.

The following tables summarize the results of this analysis for the HAQ outcomes (Table 1) and for the ACR outcomes (Table 2).

**Table 1:** Results from RR HAQ analysis: Mean estimate with 80% credible intervals for each pair-wise comparison. Significant results are marked with *. Outcome measures are % improvement for continuous HAQ and RRs for HAQ 20 and HAQ 50.

| Comparison (A vs. B) | HAQ | HAQ20 | HAQ50 |
| --- | --- | --- | --- |
|  | **Mean (80% CrI)** | **Mean (80% CrI)** | **Mean (80% CrI)** |
| Ada vs. Placebo | **0.20 (0.17,0.23)*** | **1.6 (1.4, 1.8)*** | **2.3 (1.7, 3.0)*** |
| Inf vs. Placebo | **0.10 (0.06,0.15)*** | **1.2 (1.0, 1.4)*** | **1.6 (1.0, 2.5)*** |
| Eta vs. Placebo | **0.31 (0.25,0.37)*** | **1.7 (1.4, 2.1)*** | **2.5 (1.4, 4.1)*** |
| Gol vs. Placebo | **0.23 (0.17,0.29)*** | **1.7 (1.3, 2.1)*** | **3.1 (1.6, 6.0)*** |
| Cert vs. Placebo | **0.25 (0.22,0.29)*** | **1.8 (1.6, 2.1)*** | **3.4 (2.2, 5.0)*** |
| Inf vs. Ada | **-0.10 (-0.15,-0.05)*** | **0.8 (0.6, 0.9)*** | **0.7 (0.4, 1.2)** |
| Eta vs. Ada | **0.11 (0.04,0.17)*** | **1.1 (0.9, 1.4)** | **1.1 (0.6, 2.0)** |
| Eta vs. Inf | **0.21 (0.13,0.28)*** | **1.5 (1.1, 1.9)*** | **1.6 (0.8, 3.0)** |
| Gol vs. Ada | **0.02 (-0.05,0.08)** | **1.1 (0.8, 1.4)** | **1.4 (0.7, 2.7)** |
| Gol vs. Inf | **0.12 (0.05,0.20)*** | **1.4 (1.0, 1.8)*** | **1.9 (0.9, 4.3)** |
| Gol vs. Eta | **-0.09 (-0.17,0.00)*** | **1.0 (0.7, 1.3)** | **1.2 (0.6, 2.9)** |
| Cert vs. Ada | **0.05 (0.00,0.09)*** | **1.2 (1.0, 1.4)*** | **1.5 (0.9, 2.4)** |
| Cert vs. Inf | **0.15 (0.10,0.21)*** | **1.5 (1.2, 1.9)*** | **2.2 (1.2, 3.9)*** |
| Cert vs. Eta | **-0.06 (-0.13,0.01)** | **1.1 (0.8, 1.3)** | **1.4 (0.7, 2.7)** |
| Cert vs. Gol | **0.03 (-0.04,0.09)** | **1.1 (0.8, 1.4)** | **1.1 (0.5, 2.2)** |
| sigma | **0.03 (0.01,0.05)** | **0.14 (0.01, 0.21)** | **0.43 (0.12, 0.65)** |

**Table 2:** Results from RR ACR analysis: Mean estimate with 80% credible intervals for each pair-wise comparison. Significant results are marked with *. Outcome measures are % improvement for continuous ACR and RRs for ACR 20, ACR 50 and ACR70.

| Comparison (A vs. B) | ACRcont | ACR 20 | ACR 50 | ACR 70 |
| --- | --- | --- | --- | --- |
|  | **Mean (80% CrI)** | **Mean (80% CrI)** | **Mean (80% CrI)** | **Mean (80% CrI)** |
| Ada vs. Placebo | **0.27 (0.25, 0.29)*** | **2.3 (1.9, 2.8)*** | **3.5 (2.8, 4.4)*** | **4.3 (3.1, 6.0)*** |
| Inf vs. Placebo | **0.24 (0.22, 0.27)*** | **1.9 (1.6, 2.3)*** | **2.4 (2.0, 3.0)*** | **2.7 (2.0, 3.6)*** |
| Eta vs. Placebo | **0.32 (0.28, 0.35)*** | **3.6 (2.4, 5.2)*** | **5.7 (3.0, 9.7)*** | **5.7 (2.3, 12.3)*** |
| Gol vs. Placebo | **0.25 (0.21, 0.28)*** | **1.9 (1.4, 2.5)*** | **2.9 (1.9, 4.1)*** | **3.6 (2.0, 6.2)*** |
| Cert vs. Placebo | **0.32 (0.30, 0.35)*** | **4.9 (3.8, 6.3)*** | **5.9 (4.2, 7.9)*** | **7.3 (4.4, 11.7)*** |
| Inf vs. Ada | **-0.03 (-0.06, 0.00)*** | **0.8 (0.6, 1.1)** | **0.7 (0.5, 1.0)*** | **0.6 (0.4, 1.0)*** |
| Eta vs. Ada | **0.05 (0.01, 0.09)*** | **1.5 (1.0, 2.3)*** | **1.7 (0.9, 2.9)** | **1.3 (0.5, 3.1)** |
| Eta vs. Inf | **0.08 (0.03, 0.12)*** | **1.9 (1.3, 2.9)*** | **2.4 (1.2, 4.2)*** | **2.1 (0.8, 4.9)** |
| Gol vs. Ada | **-0.02 (-0.06, 0.02)** | **0.8 (0.6, 1.1)** | **0.8 (0.5, 1.3)** | **0.8 (0.4, 1.7)** |
| Gol vs. Inf | **0.01 (-0.04, 0.05)** | **1.0 (0.7, 1.4)** | **1.2 (0.8, 1.7)** | **1.3 (0.7, 2.5)** |
| Gol vs. Eta | **-0.07 (-0.12, -0.02)*** | **0.5 (0.3, 0.8)*** | **0.5 (0.3, 1.0)*** | **0.6 (0.2, 1.9)** |
| Cert vs. Ada | **0.05 (0.02, 0.09)*** | **2.1 (1.5, 2.9)*** | **1.7 (1.2, 2.5)*** | **1.7 (0.9, 3.1)** |
| Cert vs. Inf | **0.08 (0.05, 0.12)*** | **2.6 (1.9, 3.7)*** | **2.4 (1.7, 3.5)*** | **2.7 (1.5, 4.7)*** |
| Cert vs. Eta | **0.01 (-0.04, 0.05)** | **1.4 (0.9, 2.3)** | **1.0 (0.6, 2.1)** | **1.3 (0.5, 3.4)** |
| Cert vs. Gol | **0.08 (0.03, 0.12)*** | **2.6 (1.8, 4.0)*** | **2.1 (1.2, 3.4)*** | **2.0 (0.9, 4.2)** |
| sigma | **0.03 (0.01, 0.04)** | **0.3 (0.12, 0.36)** | **0.2 (0.04, 0.35)** | **0.3 (0.00, 0.42)** |

The MTC results based on the RR scale confirm the findings from the OR scale. All differences detected by the binary HAQ and ACR measures are also detected in their continuous counterpart. More differences between anti-TNF agents were detected in the continuous analysis. This confirms the enhanced sensitivity to change of continuous measures in MTC models compared to using binary outcome measures.
